# Forest Structure and Roe Deer Abundance Predict Tick-Borne Encephalitis Risk in Italy

**DOI:** 10.1371/journal.pone.0004336

**Published:** 2009-02-02

**Authors:** Annapaola Rizzoli, Heidi C. Hauffe, Valentina Tagliapietra, Markus Neteler, Roberto Rosà

**Affiliations:** Fondazione Edmund Mach - Centro Ricerca e Innovazione, Michele all'Adige, Italy; Umea University, Sweden

## Abstract

**Background:**

The Western Tick-borne encephalitis (TBE) virus often causes devastating or lethal disease. In Europe, the number of human TBE cases has increased dramatically over the last decade, risk areas are expanding and new foci are being discovered every year. The early localisation of new TBE foci and the identification of the main risk factors associated with disease emergence represent a priority for the public health community. Although a number of socio-economic parameters have been suggested to explain TBE upsurges in eastern Europe, the principal driving factors in relatively stable western European countries have not been identified.

**Methodology/Principal Findings:**

In this paper, we analyse the correlation between the upsurge of TBE in 17 alpine provinces in northern Italy from 1992 to 2006 with climatic variables, forest structure (as a proxy for small mammal reservoir host abundance), and abundance of the principal large vertebrate tick host (roe deer), using datasets available for the last 40 years. No significant differences between the pattern of changes in climatic variables in provinces where TBE has emerged compared to provinces were no clinical TBE cases have been observed to date. Instead, the best model for explaining the increase in TBE incidence in humans in this area include changes in forest structure, in particular the ratio of coppice to high stand forest, and the density of roe deer.

**Conclusion/Significance:**

Substantial changes in vegetation structure that improve habitat suitability for the main TBE reservoir hosts (small mammals), as well as an increase in roe deer abundance due to changes in land and wildlife management practices, are likely to be among the most crucial factors affecting the circulation potential of Western TBE virus and, consequently, the risk of TBE emergence in humans in western Europe. We believe our approach will be useful in predicting TBE risk on a wider scale.

## Introduction

The relative importance of various biotic and abiotic factors in driving the emergence and spread of tick-borne diseases across Europe is currently undergoing rigorous evaluation (http://www.eden-fp6project.net/). In addition to climate change and its wide ecological implications [for a review see 1], many countries in Europe are also experiencing major socio-economic and land-use transformations. In addition to human exposure to ticks, any or all of these factors may exert a significant impact on ticks and tick host abundance and therefore on emerging zoonotic disease transmission [2], [3], [4], [5].

Tick-borne encephalitis (TBE) is a preventable zoonotic disease caused by the Western TBE virus, a species of *Flavivirus* (family Flaviviridae) with three subtypes: European, Siberian and Far-Eastern [6], [7]. The disease causes flu-like symptoms that may or may not be followed by acute meningoencephalitis and/or myelitis [8]. Disturbingly, up to one third of patients have long lasting sequelae, frequently accompanied by cognitive dysfunction and a substantial reduction in quality of life [9]; consequently, the most severely affected TBE cases have high healthcare and social costs [8]. The European subtype of the TBE virus is most often transmitted to humans by adults and nymphs of the ticks *Ixodes ricinus* and *I. persulcatus* which acquire the infection while feeding as nymphs and larvae on forest-dwelling rodents, especially the yellow-necked mouse (*Apodemus flavicollis*), which is widespread throughout the continent [10], [11], [12].

In Europe, including the Baltic States and members of the Russian Federation, TBE is an emerging public health concern, with the number of cases rising by an average of 400% over the last 30 years [13]. However, although 13 000 suspected cases of TBE are referred to European hospitals annually, in many countries the actual incidence of TBE is almost certainly underestimated, since the clinical features of milder TBE are similar to those of many other types of meningitis and/or encephalitis [14], [15]. In addition to the previously recognized risk area, which extends from eastern France to Japan between approximately 45° and 60° north latitude (Süss 2007, International Scientific Working Group on TBE: http://www.tbe-info.com/tbe.aspx), new foci have recently appeared on the northern fringes of this distribution, in Norway and southern Sweden [16]. The probability of infection is high, not only for inhabitants of endemic areas, but also for tourists travelling regularly into these regions [17].

The remarkably rapid increase in the incidence of TBE across Europe since 1974 is highly heterogeneous and there is no single explanation for this upsurge. What is known is that the risk of TBE infection in humans is dependent on the frequency of exposure to bites by infected ticks which, in turn, is dependent on human behaviour (which varies with socio-economic conditions and occupation), as well as various biotic (e. g. tick host distribution and abundance, susceptibility of hosts, proportion of immune hosts) and abiotic factors (e. g. local climatic condition and landscape features) affecting tick phenology and survival rates [3], [4], [18]. In central and eastern European countries, a combination of causes has been proposed to explain the increasing incidence of TBE [19], [5], [20]; however, most of these factors have been linked to dramatic socio-economic changes occurring in these countries since the end of Soviet rule, a situation that is not applicable to the rest of Europe.

Another major factor implicated in the increase in TBE incidence is climate change. The recent IPCC Report on Climate Change [21] outlines the wide-ranging impacts of a warming trend on the European climate, including an upward shift of the treeline and vegetational changes [1]. These generally milder climatic conditions would appear to favour TBE virus transmission especially in certain areas; in fact, in parallel with the shift of *I. ricinus* distribution to higher altitudes and latitudes, there have been reports of upward and northern shifts of TBE incidence [22], [23], [24], [25], [26], [27], [28], [29], [2], [30], [4]. Moreover, Randolph [24] has predicted that the distribution of TBE virus will be driven into higher latitudes and altitudes until 2020–2080 with extinction in southern Europe.

Despite these predictions, the changing climatic pattern does not explain all the spatial heterogeneity in increased TBE incidence across Europe [3], and observed and unexplained variance suggests that other predictors need to be considered. Forest and wildlife management are two possible factors that have been suggested [e.g. 14]. Changes in forest cover are known to have a major impact on biodiversity and ecosystem functions, [31], [32], particularly afforestation and reforestation [1]. In the case of TBE, such major ecosystem changes are likely to affect the occurrence of tick vectors and competent vertebrate hosts (*Apodemus* spp.), and it has already been reported that wildlife management practices which cause a numerical increase of few highly critical hosts within natural or anthropic ecosystems, affect the emergence and spread of vector-borne zoonoses such as borreliosis (Lyme disease) and West Nile virus [33]. However, while the effect of afforestation on tick-borne diseases, in particular on tick abundance, has been investigated [34], changes in forest structure have not.

The presence of wild ungulate species, such as red deer (*Cervus elaphus*) and roe deer (*Capreolus capreolus*), has also been shown to be essential in maintaining and amplifying tick populations and, consequently, the TBE virus [35], [36], [37], [38]. Ungulates are dilution or non-competent hosts (i.e. they act as tick hosts, but are not responsible for virus transmission between ticks); however, since adult ticks usually take their final blood meal from deer, many studies have focussed on the effect of deer exclusion on the disruption of the tick-host cycle [see 39 and references therein], which may result in a decrease or increase in transmission risk depending on the size of the exclosure [39]. However, no studies have looked at the effect of a larger-scale increase in deer abundance in combination with changes in forest structure, resulting from changes in wildlife and forest management, on TBE incidence in humans.

In Italy, TBE is a significantly less important health risk than other tick-borne diseases such as borreliosis and rickettsiosis [40]. Nonetheless, the first known Italian focus was identified in the Region of Tuscany [41], with increasing numbers being reported annually in north-eastern Italy since 1992. As in the rest of Europe, mean climatic conditions have changed considerably in Italy during the last two decades; however, in the Alps, the increase in temperature has been higher than the global mean since 1980, especially since 1985 [42], [43]. Moreover, although socio-economic conditions have changed relatively little compared to eastern Europe, forest and wildlife management has changed significantly, as a result of new regional laws. Therefore, the aim of the present study is to provide, for the first time, a review of the incidence of TBE in northeastern Italy, and to understand the relative contribution of changes in climate, forest structure and management, and large ungulate abundance to the rapid increase in TBE cases in this area.

The hypothesis tested in this paper is that substantial changes in land and wildlife management practices which improve habitat suitability for the principal TBE reservoir hosts (small mammals), and increase the number of non-competent tick hosts (roe deer) are among the most crucial factors driving the circulation potential of TBE virus and, consequently, the pattern of TBE emergence in humans. For this purpose we analysed the upsurge of TBE in a series of alpine provinces in the northern Regions of Italy using national and regional datasets available for meterological measurements, forest cover and roe deer abundance for the last 40 years. We propose this approach as a predictor of TBE qualitative spatial risk in Europe.

## Materials and Methods

### Epidemiological data

**Figure 1:**
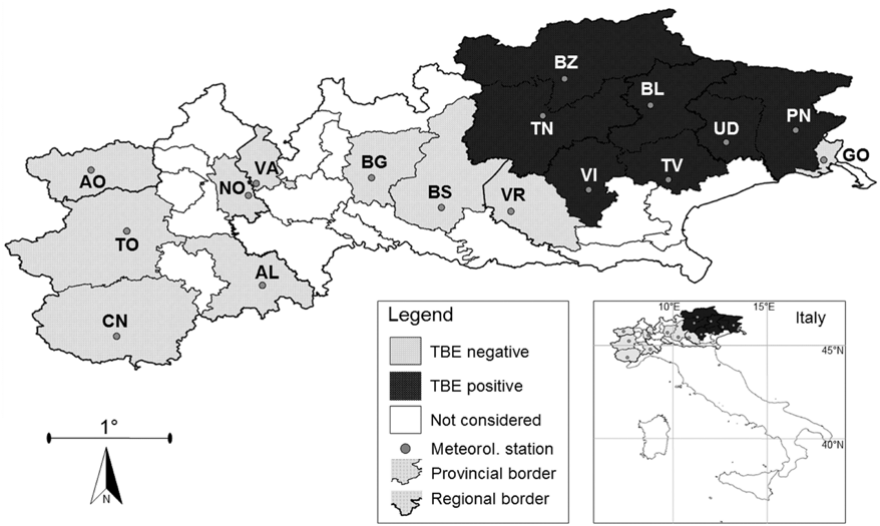
TBE-positive and TBE-negative provinces in northern Italy. (AL = Alessandria; AO = Aosta; BG = Bergamo; BL = Belluno; BS = Brescia; BZ = Bolzano; CN = Cuneo; GO = Gorizia; NO = Novara; PN = Pordenone; TN = Trento; TO = Torino; TV = Treviso; UD = Udine; VA = Varese; VI = Vicenza; VR = Verona).

Public Health Authorities, medical research institutes and hospitals from six northern Italian regions (Valle d'Aosta, Piemonte, Lombardia, Trentino Alto-Adige, Friuli-Venezia Giulia, Veneto; Fig. 1) provided the number of confirmed TBE cases from 1992 (the year of the first reported TBE case in this area) to 2006 by means of a questionnaire. Within these, 17 provinces were selected for study as potentially favourable for the emergence of TBE (i.e. alpine provinces with large expanses of forest and pasture, ecotonal areas and the presence of wild ungulates) and on the basis of availability of meteorological data. Medical experts were requested to list confirmed cases of TBE as defined by Kunz [44]: “a febrile patient with clinical signs or symptoms of meningitis or meningoencephalitis, mild to moderate elevation of cerebrospinal fluid cell count, and the presence of serum IgM and/or IgG antibodies against the TBE virus.”

Data on the presence and prevalence of the TBE virus in ticks in all of these provinces was taken from previous publications [35], [45]. Provinces with TBE clinical cases were considered TBE-positive. For each patient, province of residence, age and sex, were recorded when possible. To calculate the incidence of TBE per 100 000 inhabitants, human population size per province was obtained from the official national census data published by the National Institute of Statistics (ISTAT, Rome, Italy; http://www.istat.it).

### Vertebrate host and forest cover data

The density (number of individuals/km^2^) of red deer and roe deer per province was supplied by the Italian Ungulate Database maintained by the Istituto Superiore per la Protezione e la Ricerca ambientale (ISPRA, Bologna, Italy). The only recent and standardised data set available was relative to the period 1996–2000 [46]. Temporal changes in roe deer abundance in northern Italy were extracted from [ref. 35].

Data on forest and pasture cover and forestry management criteria per province were available from ISTAT censuses available on a 10-yearly basis from 1950 to 1990, and from the Italian National Forestry Inventory for 1997 and 2002. Forests were defined as coppices (small areas of broad-leaved forest harvested regularly at short intervals for firewood) or high stand forests (larger areas of single-stemmed coniferous, broad-leaved or mixed species grown from seed or transplanted to produce timber or stabilize soil). For forest composition, the following explanatory variables were used for statistical purposes (using annual data for all the available years): i) ratio of coppice to high stand forest cover (cop.hfor) and ii) ratio of coppice to mixed forest cover (cop.mix), since both these ratios give an indirect measure of forest seed productivity.

### Climatic data

The meteorological data for this study were obtained from the European Climate Assessment and Dataset project (ECA&D, http://eca.knmi.nl/); the Global Summary of Day (GSOD; NOAA National Data Centre Climate Data, http://www.ncdc.noaa.gov/oa/mpp/freedata.html); and from the provincial meteorological stations listed in Table 1.

**Table 1:** Position and classification of meteorological stations from which data were obtained for this study.

Region:					
Province*	Station location or name	Degrees latitude (N)	Degrees longitude (E)	Elevation (m above sea level)	Köppen-Geiger Code**
Friuli-Venezia Giulia					
GO	Ronchi dei Legionari GSOD, 161080	45.817	13.483	12	Cfa
PN	Pordenone	45.967	12.667	23	Cfa
UD	Udine	46.067	13.250	106	Cfb
Lombardia					
BG	Bergamo Orio al Serio GSOD, 160760	45.667	9.700	237	Cfb
BS	Brescia Ghedi GSOD, 160880	45.417	10.283	97	Cfa
VA	Milano Malpensa GSOD, 160660	45.617	8.733	211	Cfb
Piemonte					
AL	Novi Ligure GSOD, 161180	44.767	8.783	187	Csb
CN	Boves	44.336	7.563	575	Cfb
NO	Novara GSOD, 160640	45.517	8.667	169	Cfb
TO	Torino/Caselle GSOD, 160590	45.217	7.650	287	Cfb
Trentino Alto-Adige					
BZ	Bolzano	46.500	11.314	243	Dfc
TN	San Michele	46.189	11.134	205	Dfb
Valle d'Aosta					
AO	Aosta GSOD, 160540	45.733	7.350	547	ET
Veneto					
BL	Agordo	46.277	12.032	578	Dfc
TV	Treviso S. Angelo GSOD, 160990	45.650	12.183	23	Cfb
VR	Verona ECAD	45.383	10.867	68	Cfa
VI	Vicenza GSOD, 160940	45.567	11.517	53	Cfb

* see Fig. 1 for abbreviations.

** Cfa: warm temperate, warm temperate, hot summeŗ Cfb: warm temperate, warm temperate, warm summer; Csb: warm temperate, summer dry, warm summer; Dfb: snow, fully humid, warm summer (however, the stations are located in the valley); Dfc: snow, fully humid, cool summer; ET: polar, polar tundra (however, the stations are located in the valley, so the classification is closer to Dfc, [75]).

Daily minimum, mean and maximum temperature and total precipitation were obtained for the period 1950–2006. To obtain a homogeneous dataset, a systematic quality control procedure was implemented using SQL (Structured Query Language) in PostgreSQL DBMS (PostgreSQL Global Development Group 2007) and R Language and Environment for Statistical Computing (R Development Core Team 2007); that is, temperature data for a particular day were removed if mean temperature was less than minimum temperature; precipitation data were removed if negative values were detected or daily precipitation was greater than twice the annual value. This data filtering was performed on all data sets. For the GSOD data, which included the number of observations per day, days were removed if 50% of observations were lacking. In addition, all duplicate entries, and months or days with no records were removed from some series. After the application of the data quality assessment, a subset of all the available stations was obtained (see Table 1). Also following quality assessment, total precipitation, mean daily minimum temperature, mean daily maximum temperature were calculated: i) for each year; ii) for the 1^st^–3^rd^ decades of February, March and April, as proposed by Sumilo and colleagues [3].

### Statistical analysis

To assess possible differences in the trends of annual and decadal (10-day) climatic variables, an analysis of covariance (ANCOVA) was performed for the data for each province using the occurrence of TBE cases as a covariate.

To analyse the relationships between the occurrence of TBE clinical cases per province, forest composition and ungulate density, a Generalized Linear Model (GLM) with binomial error distribution was performed using S-PLUS 7.0 (Insightful, Seattle, Washington, USA). In this analysis, the presence or absence of clinical cases of TBE in humans in each province during the period 1992–2006 was used as the response variable, while the regional forest composition ratios (cop.hfor and cop.mix) as well as the mean density of roe and red deer were selected as fixed explanatory variables. The response variable was modelled for dependence on predictor variables using the model selection method based on the Akaike's Information Criterion (AIC) corrected for small sample size, AICc [47]. From the AICc differences (ΔAICc), we calculated AICc weights (ώ) and the relative evidence ratios. When differences in AICc values were less than two, indicating an approximately equal parsimony of models, we ranked all variables considered in the full model according to their importance [predictor weights ω_+_(*j*)].

## Results

Between 1990 and 2006, in seven out of seventeen north-eastern Italian provinces (Trento, Bolzano, Belluno, Pordenone, Udine, Treviso, Vicenza), TBE has emerged clinically (Fig. 1), with a total of 198 confirmed human TBE cases (Table 2). Over the same time period, TBE virus has also been isolated from questing ticks in most of these provinces [35], [45]. In the remaining ten provinces (Alessandria, Aosta, Bergamo, Brescia, Cuneo, Gorizia, Novara, Torino, Varese, Verona) no clinical cases of TBE or TBE virus isolation form ticks were recorded (Fig. 1).

**Table 2:** Clinical statistics from the TBE-positive provinces of northern Italy (see also Fig. 1).

Province	Nr. of cases	Mean annual incidence (nr. of cases/100 000 inhabitants)	Year of first case
Bolzano	5	0.15	2000
Trento	44	0.60	1992
Pordenone	4	0.23	2001
Udine	17	0.36	1998
Belluno	102	3.71	1994
Treviso	22	0.33	1999
Vicenza	4	0.05	1998
TOTAL	198	0.99	1992

**Figure 2:**
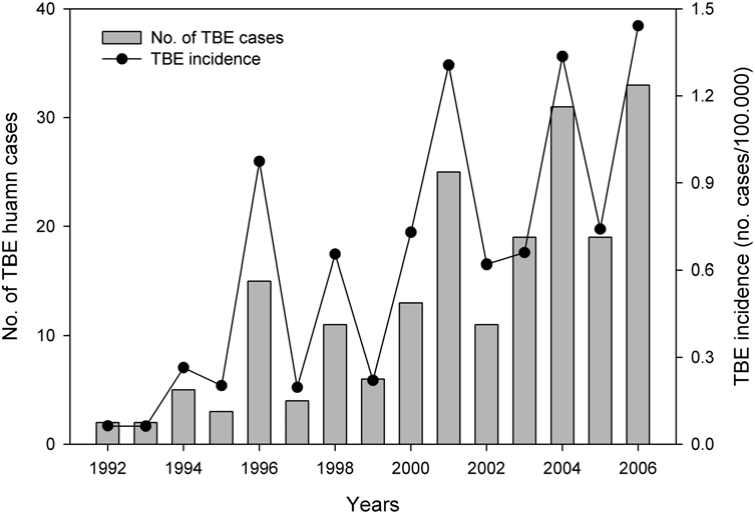
TBE incidence. Mean annual TBE incidence (number of cases/100 000 inhabitants) and annual TBE confirmed human cases in the TBE-positive provinces.

Since 1992, the mean annual incidence of human cases of TBE in TBE-positive provinces has increased steadily (Fig. 2), from 0.06 per 100 000 inhabitants (2 cases in a population of 3 398 439 in 1992) to 0.88 per 100 000 inhabitants (33 cases out of 3 739 404 in 2006). The majority of cases are concentrated in the second time period from 2000–2006 (151 cases out of 198, or 76.3%). The province with the highest total number of human TBE cases was Belluno in the Region of Veneto (Fig. 1, Table 2).

**Figure 3:**
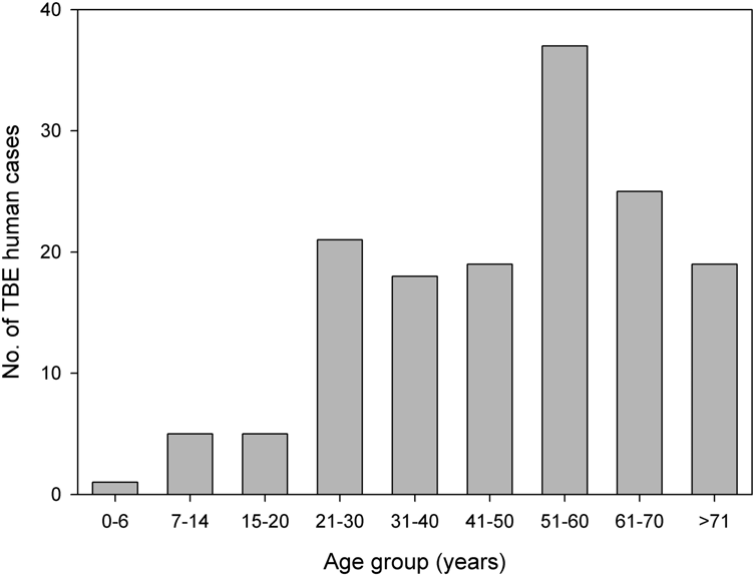
TBE human cases aggregated per age class.

The sex of patients was available for 148 out of 198 confirmed TBE cases, showing that the majority of TBE cases were male (104/148 or 70.3%). The age of patients was available for 150 TBE cases: the youngest patient was 5 years old and the oldest patient, 79. However, children (age 0–14) represented only 4.0% of total cases (6/150), while the age group with the highest incidence of TBE occurred in patients more than 50 years old (37/150 or 24.7%; Fig. 3).

**Figure 4:**
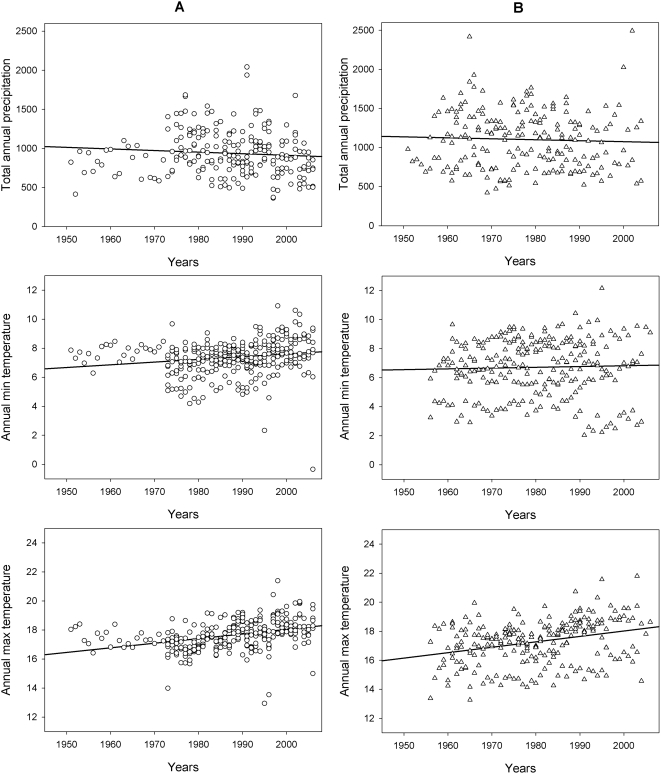
Trends in climatic variables. Annual total precipitation (top), annual minimum (middle) and maximum (bottom) daily air temperature in the TBE-negative provinces (panels A) and TBE-positive provinces (panels B) in northern Italy from 1950 to 2006 (see also Fig. 1).

From 1950 to 2006, in all 17 alpine provinces considered here, there was a significant increase in both the annual minimum temperature (ANCOVA, *F*_1,531_ = 11.73, P<0.001) and annual maximum temperature (ANCOVA, *F*_1,537_ = 56.62, P<0.001), and a significant decrease in the total annual precipitation (ANCOVA, *F*_1,425_ = 9.68, P<0.01; see slope coefficients in Table 3). However, no significant differences were observed in the trends of annual climatic variables between TBE-positive and TBE-negative provinces (Table 3 and Fig. 4). Analogously, no differences were observed in the trends of any decadal (10-day) climatic variables between positive and negative provinces.

**Table 3:** Linear regression slopes of the temporal trends for annual climatic variables using the occurrence of human TBE cases as the covariate (see also Fig. 4).

Climatic variable	Coefficients§	Value	Std. Error	t value	Pr (>|t|)
Annual total precipitation	All	−3.814	1.226	−3.112	**
	Pos	−1.477	2.027	−0.729	0.467
	Neg	−2.129	1.600	−1.331	0.185
	Diff.Pos.Neg	0.652	2.568	0.254	0.800
Annual min temperature	All	0.019	0.006	3.424	***
	Pos	0.005	0.010	0.535	0.593
	Neg	0.018	0.006	3.032	**
	Diff.Pos.Neg	−0.013	0.011	−1.134	0.257
Annual max temperature	All	0.034	0.005	7.524	***
	Pos	0.037	0.008	4.448	***
	Neg	0.028	0.005	5.499	***
	Diff.Pos.Neg	0.009	0.009	0.938	0.349

§ All, slope for all provinces pooling data; Pos, slope for positive provinces; Neg, slope for negative provinces; Diff.Pos.Neg, difference in slopes between positive and negative provinces.

** P≤0.01.

*** P≤0.001.

Roe deer density was 4.34 and 1.84 head per km^2^ in the TBE-positive and negative provinces, respectively, while red deer density was 0.75 and 0.28 head per km^2^ in the TBE-positive and negative provinces, respectively.

**Figure 5:**
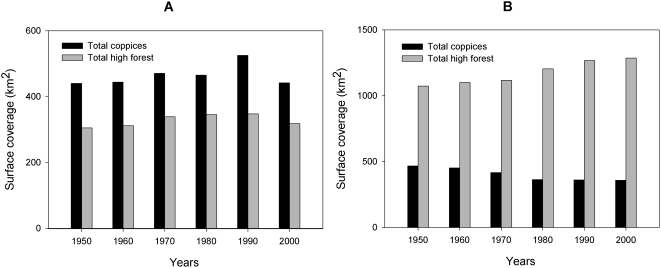
Forest variables. Total coppices and high forest surface coverage recorded in TBE-negative provinces (panel A) and TBE-positive provinces (panel B) (see also Fig. 1).

In the Italian provinces studied here, the total forest cover increased by 2.2%, from 1 702 698 to 1 741 094 ha from 1950 to 2002. Coppice surface decreased by 11.8% (from 742 582 to 663 614 ha), while high stand forest increased by 10.8% (from 960 116 to 1 077 480 ha). In TBE-positive provinces, high stand forest cover is currently higher than that of coppices (729 471 ha and 204 575 ha, respectively, accordingly to the last census in 2002). Moreover, high stand forest cover increased over the period for which data are available (1950–2002), while coppice cover decreased. Instead, in TBE-negative provinces, present coppice cover is higher than that of high stand forest (459 040 ha and 348 009 ha, respectively, in 2002) and no trends were observed for the years 1950–2002 (Fig. 5). In both TBE-positive and TBE-negative provinces, coniferous high stand forest was more widespread than broad-leaved and mixed forests: in the TBE-positive provinces, coniferous stands increased by 1.8% from 1950 to 2002, but broad-leaved and mixed stands increased their surface areas by 34.4% and 65%, respectively. In TBE-negative provinces, broad-leaved high stand forests increased by 39.9% over the same time period, while coniferous and mixed stands increased by 8.8% and 3.6%, respectively. Relative changes in pasture cover between TBE-positive and negative provinces between 1950 and 1990 were not significant.

**Figure 6:**
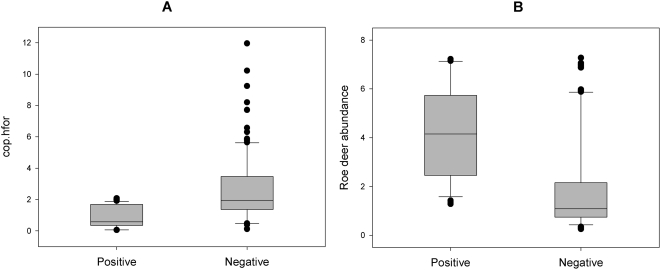
Wildlife variables. Boxplot of mean values of coppice to high forest ratio (cop.hfor) (panel A) and roe deer abundance (panel B) in TBE-positive and TBE-negative provinces of northern Italy (see also Fig. 1).

The best model for explaining the increase in incidence of TBE in humans on a provincial scale included the ratio of coppice to high stand forest (cop.hfor) and the density of roe deer (Table 4). The contribution of deer densities as predictor variables for TBE occurrence was higher for roe deer (predictor weight = 0.83) than for red deer (predictor weight = 0.45), while the contribution of forest composition was higher for cop.hfor (predictor weight = 0.91) than for cop.mix (predictor weight = 0.35). Specifically, GLM model coefficients showed a significantly negative correlation between presence of clinical TBE cases with cop.hfor (Estimated coefficient = −1.31, Std.Error = 0.68, Chi-square_1,22_ = 6.23, p = 0.012), with higher values in TBE-negative provinces (Fig. 6) and a positive, but not significant, correlation with roe deer density (Estimated coefficient = 0.43, Std.Error = 0.27, Chi-square_1,22_ = 2.57, p = 0.109) in TBE-positive provinces (Fig. 6).

**Table 4:** Akaike's Information Criterion (AICc) ranking of *a priori* models used to estimate dependence of tick-borne encephalitis (TBE) human cases on vertebrate host and forest cover parameters.

Model structure§	Df	AICc	ΔAICc	ω*_i_*	Evidence ratio
TBE∼cop.hfor+roe.deer	3	22.66	0.00	0.35	1.00
TBE∼cop.hfor+roe.deer+red.deer	4	24.00	1.34	0.18	1.95
TBE∼cop.hfor+cop.mix+roe.deer	4	24.58	1.92	0.14	2.61
TBE∼cop.hfor+red.deer	3	24.84	2.18	0.12	2.97
TBE∼cop.hfor+cop.mix+roe.deer+red.deer	5	25.96	3.30	0.07	5.20

Df, Degrees of freedom; ΔAICc, difference in AICc between the best and the present model; ω*_i_*, Akaike's weight; Evidence ratios, ratio of Akaike's weights of the best and the present model. The table shows only the first five best-ranked models.

§ TBE, provinces where human TBE cases occurred; cop.hfor, density ratio of coppice to high stand forest cover; cop.mix, ratio of coppice to mixed forest cover; roe.deer, roe deer; red.deer, red deer density.

## Discussion

This study was designed to gain insight into the contribution of changes in forest structure, deer density and climate change to the emergence of TBE in 17 alpine provinces in northern Italy since 1992.

In this first detailed review of this TBE upsurge, we have shown that out of 17 alpine provinces considered to include the most ideal habitat for the emergence of this tick-borne disease, TBE has clinically emerged in just in seven of these (Fig. 1, Table 2). In the intervening years between the first reported case in 1992, the mean annual incidence of TBE in TBE-positive provinces has increased steadily from 0.06 in 1992 to 0.88 in 2006 (Fig. 2). This upsurge is not as dramatic as that noted in the Baltic States over a similar time period (from 0.5 to 54/100 000 inhabitants [3], and current incidence is considerably lower and more patchy than some northern and central European countries (e.g. Germany: 19.1/100 000 inhabitants in 2005, [ref. 44]; Czech Republic c.10/100 000 in 2006, Slovakia c. 18/100 000 in 2006, [ref. 20]). Nonetheless, the trend in northern Italy is worrying, especially since preventive medical measures, such as vaccination of high-risk groups and an information campaign, were implemented immediately following confirmation of the first clinical case.

The risk of contracting TBE in Italy is both age (24.6% of patients are more than 50 years old; Fig. 3) and sex (70.2% are men) dependent, as previously noted by Süss and colleagues [48] for neighbouring Austria, but in contrast to reports from eastern Europe and Russia where children can be the most affected age class [49], [50]. These results may not depend so much on differences in susceptibility as on exposure to infected ticks as a result of age and sex variation in occupation or leisure pursuits; however, our present dataset does not allow us to distinguish between these two possibilities.

As outlined in the Introduction, a shift of TBE foci toward higher latitude and altitude in connection with the general warming of the European climate has been forecasted by several authors [24], [51], [52]; however, our results showed no correlation between the heterogeneous emergence of TBE in north-eastern Italy and changes in a series of climatic variables from 1950 to 2006 (Fig. 4). This result is perhaps not surprising since climate change is occurring uniformly on a much larger-than-provincial scale. In fact, comparable trends in temperature increase and precipitation decrease as seen in our study have already been confirmed in other Italian/Alpine time series (based on climatological normals from 1961–1990, Fig. 2.2 in [ref. 42]; based on averages from 1901–2000, Fig. 19 in [ref. 53]). Our conclusion also corroborates that of other groups who noted that climate change is only one of several factors driving the upsurge of TBE in other regions of Europe (the Baltic Region, [ref. 3], and central and eastern European countries, [ref. 20]).

While marked socio-economic changes following the end of Soviet rule have been pinpointed as the major causal factors for the TBE upsurge in the eastern and Baltic regions of Europe [3], [20], these factors cannot be responsible for the heterogeneous increase in TBE incidence in the rest of Europe. Instead, in Italy, some key vertebrate tick hosts, especially red deer and roe deer, have undergone equally dramatic, but more heterogeneous changes in density since the 1950s due to regional and/or provincial variation in the creation of parks and/or harvesting pressure. At the end of World War II, these two ungulate species were almost extinct in the Italian Alps, but their ranges and population sizes have been increasing ever since [46]. For example, in the Province of Trento where the longest dataset is available from the hunter's association, both roe and red deer have increased their densities more than one order of magnitude in the last fifty years (by c. 2000% and 5000%, respectively, [ref. 35]). In fact, even during the relatively short time period from 1996 to 2000 for which standardised density data were collected by Pedrotti and colleagues [46], densities of both species increased by 10%. The near-significant correlation between increases in roe deer density and incidence of TBE in the alpine provinces of north-eastern Italy partially supports our hypothesis that large vertebrate hosts could play a crucial role in TBE upsurge. For this reason, we would like to retest this hypothesis with a longer time series for all provinces. In addition, since it has been shown previously that localized absence of deer increases the probability of a TBE hotspot [39], it would be interesting to investigate if increasing deer density leads to certain changes in population dynamics (such as locally very high densities) which encourage new foci (‘hotspots’) of tick-borne disease transmission.

In addition to decisive, albeit non-uniform, changes in wildlife management across the northern Italian alpine provinces, forest management has also faced important philosophical and methodological changes, passing from the pre-19^th^ century concept of a forest as a wood-producer, to that (beginning in the 1960s) of a complex ecosystem, highly connected with the territory where it is located, and with landscape, requalification, cultural, aesthetic, hydrogeological, soil protection, and biodiversity conservation functions. Most importantly, the management of high stand woodlands is now based on an uneven-age model. Our analysis of forestry data confirms that in northern Italy, especially in the eastern regions, most of the coppices are being converted into high stands forests, except where local communities continue to collect firewood.

Interestingly, our analyses also indicated a significant correlation between an increase in TBE incidence and an increasing ratio of high stand forest to coppice cover (Fig. 6). This result may reflect the impact of an increase in high stand forests with a more natural age-distribution of trees on the distribution and abundance of the TBE virus reservoirs and vectors, more specifically, micromammals. In fact, the forest composition ratios used on our model were specifically chosen to represent habitat suitability indeces for *A. flavicollis*, the species mainly responsible of the non-viraemic transmission of TBE virus to ticks [54], [55], which is generally described as a species of mature forests with understory [56], [57]. It is widely acknowledged that the structure and species composition of vegetation in the different horizontal layers within a woodland have a considerable influence on small mammals [57], [58], [59], [60], and the episodic and synchronous production of seeds (mast) which occurs in high stand forests, but very rarely in coppices, could also have a profound influence on these small rodent populations [61], [62], [63], [64], [but see 65]. It could be argued that increasing forest cover actually reflects an increase of the TBE-vector tick, *I.ricinus*, which also prefers woodland habitat with an understory that offers favourable microclimatic (especially humidity) conditions [66]; however, while previous studies have shown that co-feeding of larvae and nymphs on rodent hosts, which is essential for TBE virus amplification, increases with tick density, tick abundance only explains the focal distribution of TBE, but not TBE incidence [11], [3]. In addition, although ungulate populations are also affected by changes in high stand forest cover, our model does not show an interaction between roe deer density and forest composition ratios. Therefore, the most likely explanation for the significant correlation between TBE incidence and high stand forest cover is the parallel increase in habitat suitability for small mammals, especially *Apodemus* spp.

While climate change may affect the future large-scale shift in *I. ricinus* distribution and the probability of TBE persistence at the extreme edges of its range as predicted by Randolph [24], this and other recent studies [e.g. 3], [71] confirm that climate change will probably not be the major causal factor of the TBE upsurge in eastern and central Europe or the Baltic States, where socio-economic factors dominate, or in alpine areas of the continent where the expansion of forests and a concomitant increase in density and distribution of rodent and wild ungulate hosts are more important factors driving up the density of ticks and their vectors.

It now appears unequivocal that in the case of TBE, accurate and early risk assessment, which is crucial for predicting the potential risk to human and animal health and for developing adaptation strategies and preventive medical services, should be based on a multidisciplinary risk assessment approach (see also http://www.thelancet.com/journals/laninf/section?issue_keyS1473-3099280829X7008-2&sectionNewsdesk **)**. We propose that the approach used in this paper is particularly useful in this regard. Such strategies are critical to European marginal or protected economies that rely on tourism and recreational activities as important sources of income, and on the public image of the natural areas and higher altitudes as ‘healthy’ and safe environments, a perception that is increasingly threatened by emerging zoonoses [72]. However, as demonstrated by our dataset, while time series of climatic data are often long and detailed, information on other biotic factors such as forest cover and wildlife abundance are frequently in the form of multi-year censes, incomplete or absent. In addition, while the standardisation of clinical case definition and diagnostic procedures has been promoted by the Italian Ministry of Health since 1995 [73], as pointed out by Sumilo and colleagues [2], the heterogeneous efforts of many other national health authorities to monitor the occurrence of TBE still impede efforts to pinpoint causal factors on a more European-wide basis. Therefore, improved data recording of biotic and clinical data are urgently required for developing accurate models of risk assessment within an eco-health perspective.

Finally, there are continuing, but as yet unheeded, calls for national vaccination programmes in endemic areas [see 13], [74], and where single TBE cases are being increasingly reported from so-called low risk areas [71]. Given the devastating physical effects of TBE and lack of a specific treatment to rid patients of the virus, the availability of an effective vaccine, and the number of recent papers reporting increases in this disease throughout the distribution area of tick vectors, our results cannot but underline the urgency of this plea.
